# Exploring Aroma and Flavor Diversity in *Cannabis sativa* L.—A Review of Scientific Developments and Applications

**DOI:** 10.3390/molecules30132784

**Published:** 2025-06-28

**Authors:** Kacper Piotr Kaminski, Julia Hoeng, Kasia Lach-Falcone, Fernando Goffman, Walter K. Schlage, Diogo Latino

**Affiliations:** 1 Independent Researcher, 2000 Neuchâtel, Switzerland; 2 Aspeya Switzerland AG, 1007 Lausanne, Switzerland; 3 Seedcraft S.L., 30510 Yecla, Spain; 4 Independent Researcher, 51429 Bergisch Gladbach, Germany; 5 Independent Researcher, 6300 Zug, Switzerland

**Keywords:** aroma, cannabis, flavor, hemp, terpenes, sensory evaluation, volatile compounds

## Abstract

*Cannabis sativa* L. exhibits a complex sensory profile governed by a diverse range of volatile and non-volatile compounds. Volatile constituents—such as terpenes, aldehydes, ketones, esters, and sulfur-containing compounds—together with non-volatile taste-active molecules including flavonoids and phenolic compounds, underlie its distinctive aroma and flavor. This review examines how genetic diversity, cultivation practices, and post-harvest processing modulate the synthesis, accumulation, and chemical transformation of these metabolites in the cannabis flower. It discusses recent advancements in the extraction, identification, and quantification of these compounds, highlighting the crucial integration of chemical characterization with sensory evaluation. By synthesizing findings from advanced analytical methodologies, this review addresses the challenges and opportunities involved in defining the sensory profiles of *C. sativa* L. varieties. Drawing insights from research on other consumer plants, strategies for future innovations are outlined, including the discovery of novel aroma and flavor compounds and the development of a universal cannabis aroma and flavor wheel. This work aims to support advancements in breeding programs, enhance product quality control, and guide future research in cannabis sensory science.

## 1. Introduction

The sensory characteristics of *Cannabis sativa* L.—its aroma and flavor—are increasingly studied due to their importance in quality control, product development, and consumer experience in jurisdictions where cannabis is legally regulated [1,2]. These attributes, which are shaped by the interplay of multiple factors, represent some of the most recognizable and influential traits of cannabis [3,4]. Genetics [5,6,7], cultivation practices [8], and post-harvest processing [9] significantly influence the accumulation of secondary metabolites, including terpenes, flavonoids, esters, aldehydes, ketones, and other aroma compounds as well as other volatile constituents [10,11,12,13,14], which collectively define the sensory profiles of different cannabis varieties. Understanding how these factors interact to shape aroma and flavor perception is essential for advancing both the consumer experience and scientific knowledge.

The sensory qualities of aroma and flavor also play an increasingly prominent role in shaping consumer preferences, supporting product differentiation in a crowded market, and are gaining attention in regulatory frameworks related to labeling and quality standards. As such, standardized approaches to measuring and describing these traits are essential not only for scientific consistency, but also for commercial and policy relevance.

In sensory science, aroma and flavor are related but distinct concepts. Aroma refers specifically to the volatile compounds perceived by the olfactory system, either orthonasally (through the nose) or retronasally (through the mouth during consumption). In contrast, flavor is a multisensory experience that integrates aroma with taste (sweet, sour, salty, bitter, and umami), mouthfeel, and thermal or chemesthetic sensations [15]. According to ISO 5492:2008 [16], flavor encompasses all sensory impressions during consumption, whereas aroma is limited to olfactory perception. In this review, we use the term aroma when referring to the olfactory properties of volatile compounds and flavor when discussing the broader sensory experience involving both taste and smell [15].

The complexity of aroma is noteworthy, as humans are capable of discerning thousands of different volatile compounds. It is significant to note that the olfactory system contributes up to 80% of what is perceived as flavor, as most nuances derive from aroma compounds [17]. Additionally, certain aroma compounds may modulate taste perception through cross-modal interactions with taste receptors. For instance, limonene imparts a citrusy aroma and may augment the perception of sweetness, whereas caryophyllene introduces a peppery, spicy note and can have a slight bitter taste [18,19].

Despite their strong connection, aroma and taste utilize distinct neural pathways, with olfactory signals processed in the olfactory bulb and taste signals in the gustatory cortex, ultimately integrating in higher brain regions to form flavor perception [18]. Understanding this relationship is essential in the science of cannabis products.

In cannabis research, it is imperative to distinguish between the subjective aroma perception of the dried plant, which is influenced by its volatile compounds at ambient conditions; the subjective aroma perception of final consumer products, such as edibles or inhaled aerosols, where processing and consumption methods alter the sensory experience; and the analytical profiles of flavor compounds in dried plant material, extracts, and aerosols, which provide objective chemical characterizations. Recognizing these distinctions is crucial for understanding how chemical compositions translate into sensory perceptions across various cannabis products.

Furthermore, the combustion of cannabis significantly alters its aroma and flavor compared to fresh plant material or vaporized extracts. While terpenes, flavonoids, and other volatile compounds contribute to the cannabis characteristics, many degrade or transform due to thermal processes [20]. Combustion generates reactive carbonyl compounds (e.g., formaldehyde, acetaldehyde, and acrolein), nitrogenous byproducts (e.g., ammonia, nitrogen oxides, and hydrogen cyanide), and volatile phenols, all of which modify or mask native aromas [21]. Additionally, cannabinoids undergo thermal degradation, influencing both flavor and psychoactive effects [22]. Unlike vaporization, which largely preserves these compounds [23], smoking introduces harsher, sometimes irritating flavor notes due to the formation of pyrolytic byproducts [24]. The method of consumption thus plays a critical role in shaping the perceived sensory characteristics of inhaled cannabis.

Accurately characterizing these sensory properties remains a significant challenge due to the complexity of the chemical interactions [3] and the presence of many compounds in minute quantities [25,26]. Advanced extraction and quantification techniques are critical for identifying and profiling the diverse metabolites responsible for cannabis sensory attributes [27,28]. These methods enable researchers to capture subtle but impactful contributors to aroma and flavor, ensuring robust chemical characterization. Standardizing such techniques is crucial for ensuring consistency and accuracy in the sensory evaluation of cannabis products, a key requirement for industry advancement.

Insights from other plant-based industries, such as hops [29], wine [30], coffee [31], tea [32], and tobacco [33], provide valuable frameworks for studying and standardizing the cannabis aroma and flavor. These industries have successfully utilized tools like aroma and flavor wheels to communicate sensory properties—primarily olfactory in nature, but also integrating consumer-perceived flavor when applicable to communicate sensory properties and enhance product differentiation [34,35,36,37,38]. Developing a universal cannabis aroma and flavor wheel would similarly enable consistent sensory evaluation, facilitating the creation of novel, uniquely scented cannabis strains in plant breeding programs, improving aroma characteristics by special cultivation techniques, and supporting product development and quality assurance.

This review provides a comprehensive exploration of the compounds responsible for cannabis aroma and flavor, focusing on their biosynthetic pathways, the influence of genetic and environmental factors, the role of advanced measurement techniques, and the lessons drawn from other industries. Furthermore, the challenges and opportunities in advancing the sensory and chemical characterization of *C. sativa* L. are highlighted, paving the way for the introduction of innovation in plant breeding, cultivation, processing, manufacturing, and product design.

## 2. Biochemistry and Genetic Regulation of Aroma and Flavor Compounds in *C. sativa* L.

The sensory diversity of *C. sativa* L. arises from a complex interplay of phytochemicals, including terpenes [13], flavonoids [12,39], and other volatile and non-volatile compounds [25,26]. These compounds not only define the aroma and flavor profiles of cannabis, but also influence consumer preferences [1] and potentially contribute to therapeutic effects [11]. The intricate interactions among these metabolites create the unique sensory complexity that characterizes different cannabis varieties.

**Figure 1:**
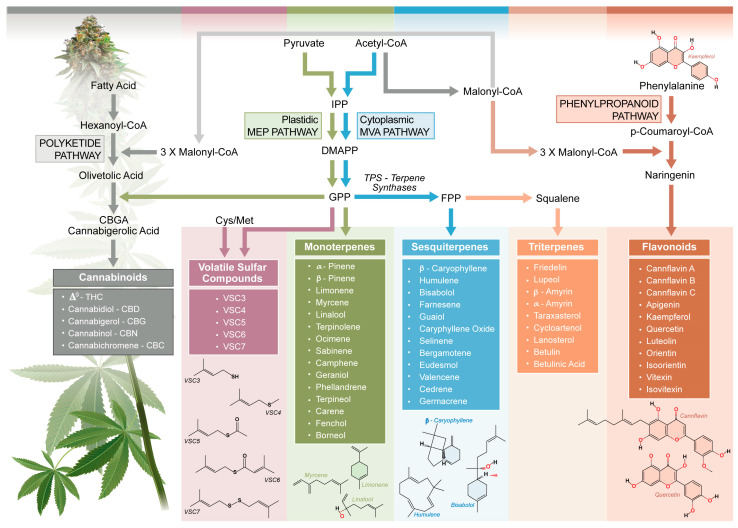
Interconnections of biosynthesis pathways of *Cannabis sativa* L. aroma and flavor compounds. The polyketide, MEP, MEV, and phenylpropanoid pathways are represented and comprehensive biosynthetic steps, as well as compounds, are included. CBC, cannabichromene; CBD, cannabidiol; CBG, cannabigerol; CBGA, cannabigerolic acid; CoA, coenzyme A; DMAPP, dimethylallyl pyrophosphate; FPP, farnesyl diphosphate; GPP, geranyl diphosphate; IPP, isopentenyl diphosphate; MEP, methylerythritol phosphate; MVA, mevalonate; TPS, terpene synthase; VSC, volatile sulfur compound. Adapted and synthesized from: Booth et al., 2020 [5]; Zager et al., 2019 [46]; Allen et al., 2019 [47]; and Flores-Sanchez and Verpoorte, 2008 [48].

The biosynthesis and accumulation of cannabinoids, terpenes, and flavonoids in *C. sativa* L. (Figure 1) are highly tissue-specific, with the highest concentrations found in the glandular trichomes of female flowers [40]. These specialized structures, particularly those on the bracts and calyces, serve as the primary sites for the production and secretion of resin containing these bioactive compounds. The small leaves surrounding the flowers, known as sugar leaves, contain moderate amounts of phytochemicals due to the presence of trichomes, whereas the larger fan leaves have mostly non-glandular trichomes, contributing minimally to cannabinoid synthesis, but instead serving as secondary sources of flavonoids [39,41]. The stems and stalks contain few glandular trichomes, resulting in low levels of these metabolites, although they may retain trace amounts of certain terpenes and flavonoid derivatives. In contrast, roots lack trichomes entirely and do not actively produce cannabinoids or terpenes, though they may contain other secondary metabolites with potential biological activity [41]. This tissue-specific distribution plays a crucial role in determining the phytochemical composition of different plant parts.

Advancements in cannabis genomics have greatly enhanced biochemical research in this area by elucidating the genetic basis of cannabinoid and terpene biosynthesis. Early efforts, such as the 2011 draft genome of *C. sativa* L. [42], provided foundational insights, while in 2020, the high-quality reference genome of wild *C. sativa* by Gao et al. [42] and another by Braich et al. [43] refined our understanding of its genetic architecture [44]. In 2024, Ryu et al. published a chromosome-level haploid assembly of *C. sativa* L. cultivar Pink Pepper, improving genome resolution and enabling precise gene annotation [45].

These genomic resources assist in exploring genetic diversity, improving breeding programs for optimized biochemical profiles, and supporting integrative studies combining transcriptomics, proteomics, and metabolomics. By studying flower terpene, flavonoid, and volatile organic compound biosynthesis, researchers can better understand the molecular basis of cannabis sensory diversity. These findings contribute to cannabis variety development and broaden the scientific understanding of plant secondary metabolism.

### 2.1. Terpenes

Terpenes are the most prominent contributors to the aroma of cannabis, with over 100 distinct compounds identified across multiple cannabis varieties [13,49]. As volatile compounds derived from the plant’s secondary metabolism, terpenes define many of the distinctive aromas associated with cannabis [13]. Terpenes are synthesized via two main pathways: the mevalonate (MVA) pathway in the cell cytoplasm and the methylerythritol phosphate (MEP) pathway in plastids [5,48] (Figure 1). These pathways produce the isoprenoid precursors dimethylallyl pyrophosphate and isopentenyl pyrophosphate, which combine to form geranyl pyrophosphate (GPP), the key precursor for monoterpenes [46,48]. Sesquiterpenes, such as caryophyllene and humulene, are derived from farnesyl pyrophosphate (FPP), an elongated product of GPP [50]. The diversity of terpenes in cannabis is attributed to the activity of terpene synthase (TPS) enzymes, which catalyze the conversion of GPP and FPP into various terpene structures [5,46]. The *C. sativa* L. genome encodes a diverse array of TPS enzymes, with at least 30 functional TPS genes identified [47]. In 2019, Allen et al. described 55 TPSs with a genomic context and tissue-specific expression, highlighting the diversity of the TPS family in cannabis [50]. TPS enzymes are classified into different subfamilies based on their phylogenetic relationships and functional roles. This enzymatic diversity allows for the generation of over 140 different terpenes in cannabis, creating a vast array of aromatic combinations [5].

Collectively, studies have demonstrated that the TPS gene family in cannabis is quite diverse, with different enzymes responsible for producing the wide array of terpenes found in cannabis resin. The expression of these genes varies between different tissues, developmental stages, and cultivars, contributing to the unique terpene profiles observed in different cannabis strains [51]. Understanding the genomic underpinnings and biochemical mechanisms of terpene biosynthesis in cannabis provides valuable insights for breeding programs aimed at developing strains with specific aroma profiles or medicinal properties. However, recent analyses of commercial cannabis samples suggest that the terpene diversity in the current germplasm may be limited, highlighting the need for the further exploration and characterization of cannabis’ genetic resources [46,50].

The most prevalent terpenes in cannabis include myrcene, which has a musky, earthy, and fruity fragrance and is associated with relaxant, sedative, anti-inflammatory, and analgesic effects [52,53]. α- and β-Limonene emit a citrus aroma reminiscent of lemons and oranges and are linked to mood elevation, stress relief, and potential neuroprotective benefits [54,55]. α-Pinene has a piney scent similar to coniferous trees and may support brain health and possess anti-inflammatory properties [56,57]. Linalool is characterized by its floral, lavender-like scent and is associated with relaxation and stress reduction [56,58]. β-Caryophyllene has a peppery aroma akin to black pepper and cloves and displays neuroprotective and antioxidant properties [59,60]. Humulene has an earthy, woody aroma and has been investigated for potential anti-inflammatory effects [61]. Ocimene, featuring a sweet, herbal, and sometimes fruity fragrance, is thought to have antiviral and antifungal properties [62,63]. Nerolidol, recognized for its woody, citrusy scent is being investigated for neuroprotective, anti-parasitic, and antimicrobial potential [64,65,66]. Terpinolene has a complex blend of floral, herbal, and citrus notes. This terpene is less common and is still under study; it may have sedative effects [67,68]. Finally, α-Bisabolol has a sweet floral aroma and is being studied for possible pharmacological benefits [69,70]. Table 1 provides a concise summary of key cannabis terpenes, including their chemical classification and characteristic aroma and flavor attributes.

### 2.2. Flavonoids and Other Aroma and Flavor Compounds

Flavonoids are synthesized via the phenylpropanoid pathway [39]. This pathway begins with the conversion of phenylalanine to cinnamic acid, which is catalyzed by phenylalanine ammonia-lyase. Chalcone synthase converts cinnamic acid into chalcones, which are precursors of flavonoids. Flavonoid synthase and other enzymes modify these chalcones into diverse flavonoid structures. Cannabis-specific flavonoids, such as cannflavins A, B, and C, contribute to aroma and flavor, with the latter primarily through taste, while exhibiting notable bioactivity, including anti-inflammatory, antioxidant, and neuroprotective properties [39]. Common flavonoids like quercetin, kaempferol, and apigenin further enhance the sensory complexity of cannabis by contributing a bitter taste.

In addition to terpenes and flavonoids, volatile organic compounds such as esters, aldehydes, and ketones play crucial roles in defining the nuanced aroma and flavor profiles of cannabis. Recent research has also demonstrated the significant role of minor volatile sulfur compounds (VSCs) and other nonterpene compounds in influencing the intricate aroma profile of *C. sativa* L. [25]. Prenylated VSCs, such as 3-methyl-2-butene-1-thiol, are mainly responsible for the skunky and pungent odor of cannabis, with their levels peaking during the late flowering and curing stages. These compounds, which are structurally analogous to bioactive sulfur molecules found in garlic, exhibit potency even at low concentrations and substantially affect the sensory characteristics of certain cultivars. Furthermore, other nonterpene volatiles, including skatole (3-methylindole) and 3-mercaptohexyl derivatives, contribute distinctive sweet, savory, or citrus-like aromas to exotic cannabis varieties [26]. These findings challenge the conventional focus on terpenes as the primary determinants of aroma, underscoring the importance of minor volatiles in shaping the aromatic complexity of cannabis.

### 2.3. Trait Associations and Exclusivity

The sensory and chemical diversity of *C. sativa* L. is shaped by the interplay of genetic, metabolic, and environmental factors. Certain traits, such as terpene and cannabinoid profiles, often occur together due to shared biosynthetic pathways or genetic linkages, while others exhibit exclusivity due to metabolic competition or genetic trade-offs [46]. Understanding these relationships is crucial for optimizing breeding strategies, enhancing desired traits, and achieving consistent product profiles.

Some compounds influencing the aroma and flavor perception are strongly correlated due to common biosynthetic origins. The sensory and chemical diversity of *C. sativa* L. is influenced by a complex interplay of genetic, metabolic, and environmental factors. Certain traits, such as terpene and cannabinoid profiles, often co-occur due to shared biosynthetic pathways or genetic linkages, while others may exhibit exclusivity resulting from metabolic competition or genetic trade-offs [46]. For example, terpenes and cannabinoids both originate from the isoprenoid pathway, and their production relies on shared precursors such as GPP. This overlap often leads to the co-occurrence of specific terpene and cannabinoid profiles [6,10]. These combinations likely arise from selective pressure during breeding programs that target complementary traits to meet consumer preferences or therapeutic needs. Understanding these relationships is crucial for optimizing breeding strategies, enhancing desired traits, and achieving consistent product profiles [10].

Empirical studies have demonstrated correlations between specific terpene and cannabinoid profiles, suggesting coordinated regulation. For instance, integrative analyses of glandular trichomes from various cannabis strains revealed co-expression networks of genes involved in both cannabinoid and terpene biosynthesis [46]. This co-expression indicates a coordinated regulation of these pathways, likely due to shared precursors and biosynthetic routes as noted above.

Additionally, variations in terpene profiles among cannabis cultivars have been linked to differences in the cannabis TPS gene family. A study characterizing five cannabis cultivars with contrasting terpene profiles identified 33 different cannabis TPS genes [6]. The variations in the cannabis TPS gene family composition and expression levels contribute to the diverse terpene profiles observed across the cannabis cultivars.

While these studies highlight the coordinated regulation of cannabinoid and terpene biosynthesis, direct evidence of metabolic competition between these pathways remains limited. Despite sharing common precursors such as GPP, the current research does not conclusively demonstrate that the biosynthesis of cannabinoids and terpenes competes for these substrates under natural conditions [71]. Further research is necessary to elucidate the extent of the metabolic competition or cooperation between these pathways.

### 2.4. Practical Implications for Breeding

The associations and exclusivity of aroma and flavor-related traits in *C. sativa* L. have important implications for breeding programs. Understanding the genetic and metabolic mechanisms underlying these relationships allows breeders to make informed decisions when selecting parent varieties.

Breeding *C. sativa* L. cultivars with an elevated terpene content while maintaining balanced cannabinoid levels necessitates a nuanced understanding of their shared biosynthetic pathways and potential metabolic interactions. Marker-assisted selection, which identifies genetic markers associated with desired traits, is a valuable tool in this process [72,73,74]. As an example, by targeting markers linked to high TPS activity or efficient cannabinoid biosynthesis, breeders can optimize multiple traits simultaneously.

Managing trait exclusivity is important for achieving consistent variety profiles. For medical purposes, varieties with high cannabidiol (CBD) and specific terpene profiles are often chosen for their therapeutic potential, whereas recreational markets may favor high tetrahydrocannabinol (THC) levels combined with unique aromatic qualities. Recognizing the balance between these traits helps ensure that breeding programs meet their intended market objectives.

Genomic and metabolomic technologies, such as clustered regularly interspaced short palindromic repeats (CRISPR)-Cas9, can clarify cannabis trait relationships and fine-tune metabolic pathways [72,75,76]. Such technologies could enable the co-expression of previously exclusive traits. Further exploration will enhance the understanding of cannabis phytochemistry and drive innovations in breeding and cultivation.

Maintaining robust agricultural properties―such as optimized nutrient and water use, temperature tolerance, pest resistance, and shortened growth cycles―remains critical even as breeders reconfigure cannabinoid and aroma profiles [77]. While modifications to flavor traits and the cannabinoid content can enhance the product quality, there is increasing evidence that these traits are intertwined with plant stress responses and the overall cultivation performance [78]. For instance, certain secondary metabolites may play roles in mediating plant defense mechanisms, potentially influencing resilience to environmental stresses. However, current breeding programs increasingly emphasize decoupling these traits to sustain or even improve core agronomic characteristics independently of aromatic and cannabinoid profiles [73,77]. This separation is essential to ensure that an enhanced flavor or potency does not come at the cost of reduced cultivation robustness, thereby enabling growers to meet both market demands and production sustainability targets.

## 3. Strategies to Influence *C. sativa* L. Aroma and Flavor Profiles

The production and accumulation of aroma and flavor in *C. sativa* L. are determined by an interplay of genetic, environmental, and post-harvest factors. By optimizing these variables, it is feasible to enhance the profiles of aroma and flavor compounds. This section examines various methods for influencing yield and profiles, ranging from genetic manipulation to post-harvest handling techniques.

Genetics plays a foundational role in determining the terpene composition and concentration. Selective breeding programs aim to enhance terpene production by targeting key genes involved in terpene biosynthetic pathways, such as those governing the MVA and MEP pathways. These pathways lead to the synthesis of monoterpenes, sesquiterpenes, and other terpene classes. Modern genetic tools, including CRISPR-Cas9 and RNA interference, have opened new avenues for precisely modifying cannabis genes [72,75,76]. By enhancing the expression of specific TPSs or suppressing competing pathways, breeders can amplify the production of desirable terpenes.

Breeding in *C. sativa* L. has evolved from unstructured selection in the illegal market to advanced genomic-driven programs. Early breeding efforts relied on uncontrolled hybridization without documented parentage, leading to diverse chemotypes with inconsistent traits [72]. Modern breeding integrates marker-assisted selection and genomic tools that are aimed at enhancing terpene and cannabinoid profiles while maintaining genetic stability [79]. These advancements can potentially enable precise cultivar development in the future, improving both commercial appeal and medicinal applications.

Environmental conditions during cultivation also have a significant impact on terpene synthesis and accumulation. Adjustments in light, temperature, soil composition, and water availability can dramatically influence the terpene profile of cannabis plants. For instance, specific light wavelengths, particularly in the blue and ultraviolet (UV) ranges, have been shown to stimulate terpene production. In a recent review, researchers explored how light manipulation can enhance the production of cannabinoids and terpenes in *C. sativa* L. by leveraging their role as photo-protectants. This study highlights that UV-B radiation increases trichome numbers and stimulates cannabinoid biosynthesis, while blue light enhances both terpene and specific cannabinoid accumulation. These findings underscore the potential of tailored light spectra, such as UV and blue-light-emitting diodes (LEDs), to optimize phytochemical yields during cultivation and post-harvest processes [77]. LED technology allows growers to precisely control the light quality and duration, enhancing the synthesis of specific terpenes.

Nutrient optimization, including the balance of nitrogen, phosphorus, potassium, and essential micronutrients, supports enzymatic activities related to terpene biosynthesis [80]. In a recent study, researchers found that high ammonium-to-nitrate (NH4/NO3) ratios negatively affect medical cannabis by reducing the yield, cannabinoids, and terpenes. This study highlights the importance of optimizing the nitrogen source for cannabis growth and phytochemical composition [81]. A recent study found that while high potassium (K) levels support cannabis growth, a K deficiency enhances cannabinoid and terpene concentrations, highlighting the need to balance the K supply for an optimal yield and phytochemistry [82]. Irrigation management further contributes, as controlled water stress can mimic natural environmental conditions, triggering the plant’s defense mechanisms and increasing terpene synthesis [83].

Elicitors, such as salicylic acid (SA), methyl jasmonate (MeJA), and ascorbic acid, significantly enhance the production of aromatic and bioactive compounds in *C. sativa* L. Studies show that SA and MeJA upregulate key biosynthetic genes, leading to increased cannabinoid and terpene accumulation in inflorescences and leaves [84]. SA has been identified as an effective hormonal regulator of promoter activity controlling the transcription of genes responsible for cannabinoid synthesis [85]. Ascorbic acid has also been shown to stimulate gene expression linked to delta-9-THC and CBD biosynthesis, boosting the levels of these metabolites [86].

Although studies of molecules with an elicitor’s effects on aroma-relevant volatiles and flavor-contributing compounds in cannabis are scarce, research on other aromatic plants with similar glandular trichomes can provide valuable insights. Plant endogenous elicitors, such as MeJA and methyl salicylate (MeSA), are well known for their ability to upregulate the secondary metabolism in plants [87]. In *Ocimum basilicum* L. (basil), an aromatic plant species with glandular trichomes structurally analogous to those in *C. sativa* L., MeJA application resulted in substantial increases in β-caryophyllene (133%), 1,8-cineole (147%), linalool (113%), and limonene (111%) compared with untreated controls [88]. Similarly, SA application enhanced the accumulation of these terpenes by a respective 45%, 53%, 65%, and 31%. These results underscore the potential of elicitor-based technologies to enhance terpene biosynthesis and accumulation in *C. sativa* L., modulating its aromatic properties and potentially its therapeutic effects.

The foliar application of SA to *Varronia curassavica* Jacq. (Boraginaceae), a Brazilian medicinal plant that accumulates essential oils in glandular trichomes, resulted in an 18% increase in its total essential oil content. Additionally, the concentrations of α-Humulene and β -caryophyllene doubled, highlighting the efficacy of SA in enhancing the biosynthesis of these key terpenes [89]. Similarly, an SA 0.5 mM and 1 mM application to peppermint (*Mentha* × *piperita*) plants increased their essential oil content by 50% and 35%, respectively. Monoterpene levels were enhanced and sesquiterpene levels were decreased. There were notable increases in 1,8-cineole (+70%), menthofuran (+30%), and isomenthyl acetate (+2020%), all of which are key contributors to peppermint’s aroma profile. Conversely, menthone (−6%), pulegone (−29%), and germacrene D (−57%) declined following the application of SA 1 mM [90].

Jasmonic acid (JA) and MeJA were reported to significantly influence the essential oil yield and composition of *Lavandula angustifolia* Mill. The application of JA 10 mM resulted in the highest percentage (0.82–0.93%) and yield (1.77–1.84 mL) of essential oil per plant. The application of MeJA 1.0 mM also enhanced the essential oil content (0.70–0.78%) and yield (1.33–1.50 mL). The application of both elicitors increased key terpenes. The levels of linalool and camphor were increased by +7.4% and +28.6%, respectively, with JA 10 mM and +6.2% and +8.5%, respectively, with MeJA 1.0 mM [91].

Beyond these examples, elicitor application in transgenic *Mentha spicata* L. (spearmint) demonstrated that the genetic background also plays a critical role in determining terpene biosynthesis. Under the elicitor of salt stress (800 nM NaCl), wild-type plants produced only monoterpenes, whereas transgenic plants expressing isopentenyl pyrophosphate isomerase (*MSIso*) or limonene synthase (*MSLimo*) accumulated additional terpenes. Following elicitation, plants expressing *MSIso* contained 15 terpenes, including phenylpropenes, while plants expressing *MSLimo* accumulated levels of sesquiterpenes exceeding 12% of the total terpenes. These findings suggest that both a favorable genetic background and elicitation are required to observe specific modifications in terpene biosynthesis [92].

Further supporting this interplay between genetics and elicitor response, intraspecific variation in *Arabidopsis thaliana* terpene emissions has been linked to allelic differences in TPS genes (TPS02 and TPS03) and their subcellular localization [93]. Elicitation with coronalon, a jasmonate mimic, induced significant variation in monoterpene and sesquiterpene emissions among accessions, demonstrating that terpene biosynthesis is shaped not only by elicitor application, but also by the genetic architecture controlling TPS activity and compartmentalization. This highlights that genetic background determines the extent to which elicitors can modify terpene profiles, reinforcing the need for an integrated approach when optimizing terpene production.

Combining these methods―genetic selection, optimized cultivation practices, and meticulous post-harvest techniques―yields the most effective results [71,94]. For example, selecting varieties with high terpene potential, cultivating them under specific light and nutrient regimens, and employing precise drying and curing methods can maximize the aroma and flavor qualities of cannabis (aroma referring to volatile compound-driven olfactory perception and flavor encompassing the broader sensory experience during use). By leveraging advances in genetics, agronomy, and post-harvest handling, it is possible to not only preserve, but also enhance the terpene profiles of *C. sativa* L., ultimately improving the sensory experience for consumers and expanding the applications in both the medicinal and recreational contexts.

## 4. Stability of *C. sativa* L. Aroma and Flavor Compounds

### 4.1. Factors Affecting the Stability of Aroma and Flavor Compounds

Protecting the stability of terpenes and volatile compounds is vital for maintaining the sensory and therapeutic qualities of *C. sativa* L. as they are sensitive to environmental conditions, some of which can lead to degradation or changes in the aroma and flavor. Understanding and enhancing preservation techniques is crucial for ensuring product quality and longevity. By developing better preservation methods and understanding environmental impacts, researchers and producers can maintain the sensory and therapeutic attributes of cannabis from harvest to consumption.

Other industrial plants can serve as a reference when considering appropriate environmental conditions for cannabis. For example, the storage conditions of hops (*Humulus lupulus* L.) play a crucial role in preserving their essential oil composition, which directly influences their aromatic profile. Studies have demonstrated that exposure to oxygen, heat, and light accelerates the degradation of volatile terpenes and essential oils, leading to a decline in the aroma intensity and quality over time. To mitigate this degradation, anaerobic (oxygen-free) storage at low temperatures is widely used to maintain the chemical integrity of hops [95]. This knowledge could be directly applied to the storage of cannabis (*C. sativa* L.), given its similar reliance on terpenes and cannabinoids for aroma.

Terpenes and other volatile organic compounds are prone to degradation because of their chemical nature and sensitivity to external conditions. Key factors affecting the stability of these molecules include light, heat, oxygen, and humidity. Regarding light exposure, UV and other light wavelengths can catalyze photochemical reactions, leading to the breakdown of terpenes and the formation of undesirable byproducts. For example, limonene can oxidize under UV exposure to produce terpinolene or other oxidized derivatives, altering its citrusy aroma. Heat is also a concern, as elevated temperatures accelerate the volatilization and degradation of terpenes [96]. During drying and curing, excessive heat can cause significant terpene loss, as these have low boiling points (e.g., myrcene volatilizes at 167 °C). Controlled temperature management during processing is crucial to minimize this effect. Oxygen exposure triggers oxidative degradation, particularly in monoterpenes like pinene and limonene. Oxidation not only reduces terpene concentrations, but also generates additional compounds with different sensory properties, such as alcohols or ketones, which can alter the aromatic characteristics and perceived flavor of cannabis products. An analysis of European CBD oils revealed notable discrepancies in the cannabinoid content, terpene fingerprints as quality indicators, and lipid oxidation profiles as markers of oil stability, underscoring the need for standardized quality regulations [97]. Finally, improper humidity levels can promote microbial growth, indirectly affecting terpene stability [98]. High humidity also accelerates enzymatic activity that can degrade volatile compounds [99].

Cannabinoids and terpenes can also undergo interconversion under specific environmental conditions, further complicating their stability [100,101]. This phenomenon is particularly pronounced during drying, curing, and storage. It has been shown that the irradiation of cannabis flowers increases the levels of some terpenes [102]. These interconversions highlight the importance of precise control over post-harvest conditions to preserve the intended sensory attributes of cannabis products.

### 4.2. Preservation Strategies

Given the volatility and reactivity of compounds influencing aroma and flavor perception, effective preservation strategies are necessary to maintain their stability and potency over time. Controlled storage conditions can help preserve the *C. sativa* L. aroma and flavor. For example, a novel packaging method using external terpenes and airtight storage demonstrated the effective preservation and enhancement of the terpene content in cannabis inflorescence [100]. Headspace gas chromatography mass spectroscopy was used to confirm improved profiles over extended durations. To properly store cannabis, it should be kept in airtight, opaque containers to minimize exposure to light and oxygen. Ideally, it should be stored under low-temperature conditions (preferably below 25 °C) with low humidity levels (approximately 50–60%), both of which help to slow down the degradation processes. Refrigeration or freezing can preserve terpenes by slowing down volatilization and chemical reactions. However, freezing must be carefully managed to avoid structural damage to cannabis trichomes, which contain most terpenes [103]. Removing oxygen from storage environments through vacuum sealing or replacing it with inert gases, such as nitrogen, can significantly reduce oxidative degradation. These methods are commonly used for long-term storage and transport.

Freeze-drying, or lyophilization, is an advanced drying technique that enhances the preservation of cannabinoids and terpenes while preventing microbial contamination. Unlike traditional drying methods, which rely on heat and extended exposure to air, freeze-drying rapidly freezes cannabis and removes moisture through sublimation under vacuum conditions. Research has demonstrated that this method significantly improves the retention of key cannabinoids such as cannabidiolic acid, cannabigerolic acid, and cannabigerol, potentially increasing their concentrations up to threefold compared to fresh plant material [104]. Furthermore, freeze-drying minimizes terpene loss, preserving the plant’s aromatic profile more effectively than conventional drying [105]. Studies using advanced analytical techniques such as solid-phase microextraction/gas chromatography/gas chromatography–mass spectrometry (GC-MS) and high-performance liquid chromatography (HPLC) confirm that freeze-drying maintains a more stable phytochemical composition, making it an optimal method for high-quality cannabis processing [106]. Considering its superior ability to retain bioactive compounds, freeze-drying is increasingly being adopted for pharmaceutical and commercial cannabis applications.

Emerging technologies such as microencapsulation or nanoencapsulation involve embedding terpenes in protective carriers like lipids or polymers. These techniques shield terpenes from environmental stressors and allow for controlled release, extending the shelf life and maintaining the aroma intensity [107]. The addition of natural or synthetic antioxidants, such as vitamin E or butylated hydroxytoluene, to cannabis products inhibits oxidative reactions, thereby preserving the terpene content and aromatic integrity [108].

## 5. Cannabis Flavor Profiling Framework

The characterization of *C. sativa* L. aroma and flavor profiles is a multidisciplinary endeavor that combines chemistry and sensory science. Establishing a standardized framework is essential for fostering consistency, accuracy, and communication across industries. Such a framework must integrate robust quantification methods, structured sensory tools like aroma and flavor wheels, and insights from related plants and industries.

### 5.1. Extraction and Quantification Methods for Aroma and Flavor Compounds

Accurately extracting and quantifying aroma and flavor compounds in *C. sativa* L. is essential for both understanding and standardizing its sensory characteristics. Cannabis extraction techniques encompass a wide range of methods. These include conventional approaches such as solvent-based extractions (e.g., ethanol and hexane) and supercritical CO_2_ extraction, and innovative techniques such as microwave-assisted extraction, ultrasound-assisted extraction (UAE), and pulsed electric fields, each of which can be tailored to optimize the recovery of cannabinoids, terpenes, flavonoids, and other bioactive compounds while preserving their biological properties [27,28,109]. Furthermore, advanced analytical techniques, such as GC-MS, gas chromatography–olfactometry (GC-O), and HPLC, are typically used to measure the volatile and non-volatile compounds that contribute to the cannabis aroma (olfactory properties) and flavor (multisensory experience) [27,110]. Here, we summarize recent research related to the extraction and quantification to highlight current scientific directions in this field.

A recent study comparing the effects of supercritical and liquid CO_2_ extraction methods on hemp seed oil demonstrated that supercritical CO_2_ extraction achieved higher oil yields (93%) and an enriched carotenoid content, while liquid CO_2_ extraction preserved more cannabinoids, alongside polyphenols and tocopherols, making both methods effective and environmentally friendly alternatives to traditional solvent-based techniques [111]. Another study coupled supercritical CO_2_ extraction with on-line fractionation to separate aroma compounds. This method effectively isolated volatile terpenes such as myrcene, limonene, and β-Caryophyllene, demonstrating its ability to selectively extract and fractionate these while preserving their quality [112]. The findings highlight the potential of supercritical CO_2_ extraction for producing high-purity terpene-rich extracts suitable for downstream quantification methods. Other studies have employed UAE to recover volatile aroma compounds from industrial hemp inflorescences. The method demonstrated high efficiency in isolating terpenes and other volatiles, offering a fast, low-temperature alternative to conventional techniques [113,114]. This highlights the potential of UAE as a sustainable and effective approach for extracting aromatic profiles from hemp.

GC-MS yields the detailed identification and quantification of volatiles, such as terpenes and esters, by separating and analyzing their molecular structures [115]. GC-O enhances this analysis by correlating specific compounds with sensory perceptions through human olfactory evaluation [14]. HPLC is typically used to measure less volatile compounds like cannabinoids and flavonoids, which also contribute to flavor and aroma profiles [115].

Standardizing quantification methods is critical for achieving consistent and reproducible data across studies and industries. Variability in sampling, preparation, and analytical protocols can lead to discrepancies in readings, which can complicate comparisons and applications. Establishing best practices for quantification—such as defining optimal extraction methods, calibration standards, and reporting conventions—ensures reliability and facilitates the creation of tools like aroma and flavor wheels, bridging subjective experiences with objective analysis.

### 5.2. Aroma and Flavor Wheel Development

The contribution of individual aroma and flavor compounds to the sensory profile of cannabis is influenced by their physicochemical properties, including volatility and concentration, as well as their interactions with co-occurring molecules [3,116]. Furthermore, interactions between terpenes and cannabinoids can modify sensory perceptions, highlighting the need for an integrative approach in profiling.

The creation of a cannabis flavor wheel would be a pivotal step in standardizing the sensory characterization of *C. sativa* L. Flavor wheels are well-established in industries such as beer, wine, coffee, tea, and tobacco, and offer a structured methodology for identifying and communicating aroma and flavor profiles [34,36,37,38,117]. A cannabis-specific flavor wheel could serve as a valuable tool for growers, researchers, and consumers by linking subjective sensory experiences with scientific analysis.

Creating a comprehensive flavor wheel involves sensory analysis by trained panels and advanced techniques to identify and measure the aroma and flavor compounds. Expert panels assess cannabis samples to build a standard lexicon of sensory attributes, ensuring consistency across varieties and products, similar to the practices in the wine, coffee, tea, and tobacco industries. Identification and quantification are crucial for linking chemical analyses to sensory perceptions. To ensure reliable sensory evaluations, panels should consist of trained assessors selected and trained according to International Organization for Standardization 8586:2023 guidelines [118], which specify criteria for the selection, training, and number of sensory assessors for representative analysis.

A universal cannabis flavor wheel would benefit growers by providing a framework for breeding and marketing targeted sensory profiles. Consumers receive a tool to match preferences with effects, while researchers benefit from a standardized system that aids data comparison and advances the scientific understanding of the cannabis aroma and flavor.

While aroma research of hops (*H. lupulus* L.) provides a well-studied reference [34] for cannabis aroma research owing to their shared Cannabaceae lineage, other plants and industries also offer valuable insights into flavor profiling. Products from both the agriculture and beverage industries, such as lavender, citrus fruits, pine trees, wine, coffee, tea, and tobacco, demonstrate overlapping chemical profiles and sensory methodologies that can enrich cannabis research. By integrating findings from related plants and industries, cannabis researchers can develop a richer, more comprehensive approach to aroma and flavor profiling. Each offers unique perspectives on terpene production, flavor stability, and sensory perception, enhancing the scientific and commercial potential of cannabis.

**Figure 2:**
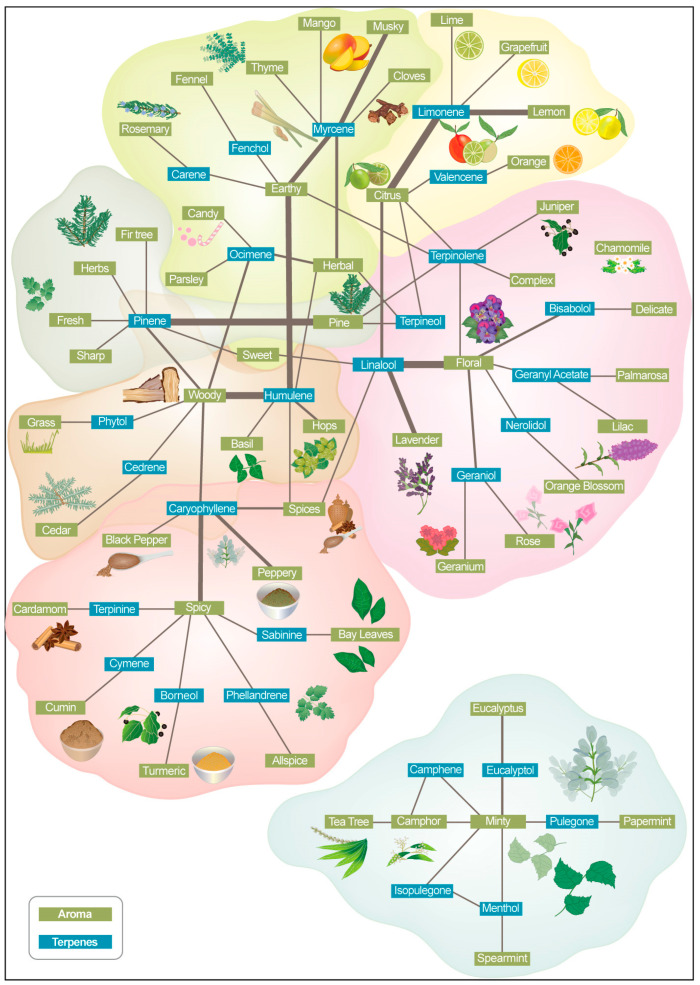
Association of terpenes and aroma descriptors in commercial cannabis products.

To visualize the relationship between cannabis aroma and flavor descriptors and commercially available terpenes that are available on the market and how they are described by producers, we created a map (Figure 2). This map incorporates aroma and terpene keywords extracted from available Aroma Wheels related to cannabis flower strains (Supporting Table S1). This comparative visualization can help to gain deeper insights into the keyword network. This integrative approach allowed for a comprehensive understanding of aroma–terpene profiles relevant to the research objectives.

**Figure 3:**
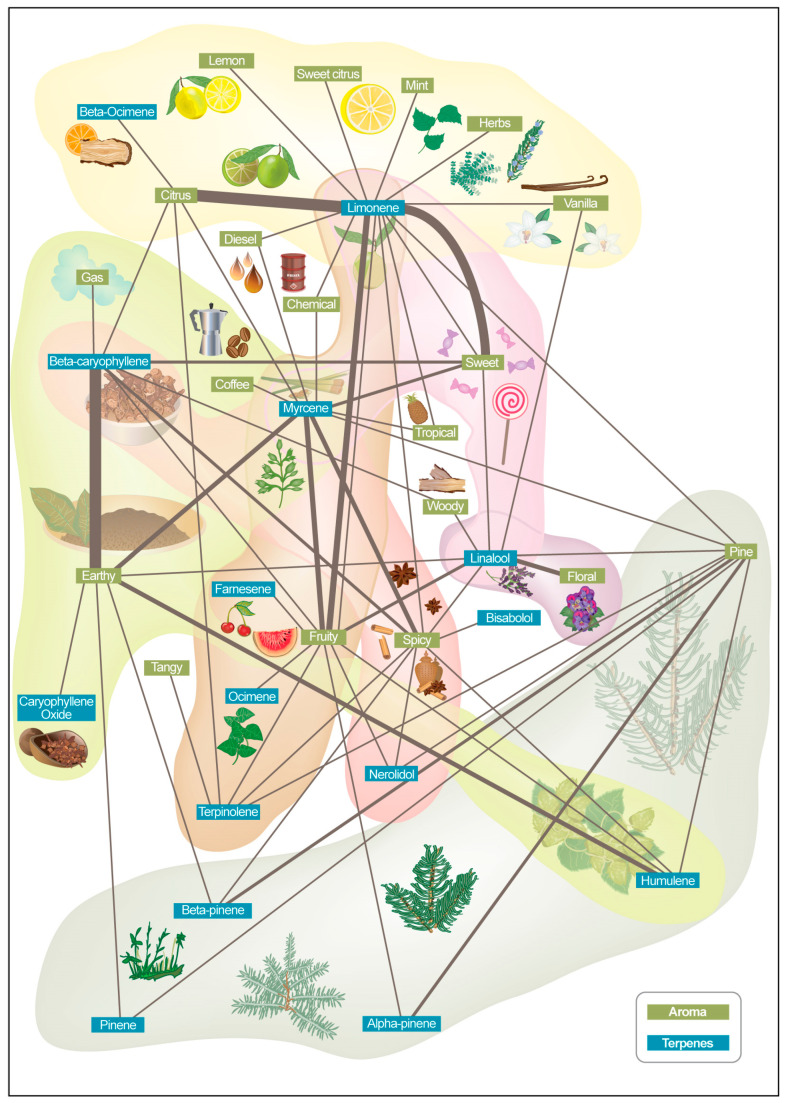
Aroma keyword network map constructed from approximately 1500 cannabis product descriptions. Data were aggregated and harmonized from various online sources, including company websites, marketplaces, and repositories. Extracted aroma- and terpene-related keywords were analyzed for co-occurrence patterns and encoded into a graph structure, illustrating the relationships and clustering of sensory descriptors across products.

Furthermore, we developed a framework to collect, analyze, and categorize aroma and terpene-related information from product descriptions. Data aggregation was performed on approximately 1500 product descriptions (Figure 3). The data were aggregated and harmonized across different sources, including information extracted from web sources of companies, marketplaces, and other repositories (Supporting Table S2). Extracted keywords were further analyzed to encode relationships and construct a graph representation of the keywords (aroma keyword network) which represent the intricate aroma and terpene interactions.

Terpenes were included in the figure due to their significant role in cannabis product descriptions. They are a primary contributor to the aroma and flavor of cannabis, alongside other compounds (Figure 1). Figure 3 identifies the most-used descriptors, the frequency of their use together, and which terpenes are commonly described by them. For example, the descriptors floral and lavender are frequently used for linalool; citrus, lemon, and orange are frequently used with limonene; pine is frequently used with pinene; earthy and woody are frequently used with humulene; and woody, spicy, and peppery are frequently used with caryophyllene.

## 6. Conclusions

The aroma and flavor of *C. sativa* L. are defining features that contribute to its identity, appeal, and potential therapeutic effects. These sensory attributes arise from a complex interplay of genetic, biochemical, and environmental factors, with terpenes, flavonoids, and other volatile compounds playing central roles. Their diversity and interactions underscore the challenges and opportunities in characterizing and standardizing cannabis sensory properties.

Advances in genetic research, sensory analysis, and metabolomics have paved the way for deeper insights into the biosynthesis and regulation of aroma and flavor compounds. By integrating findings from related plants such as hops, and leveraging tools such as flavor wheels, the cannabis industry can develop standardized approaches to sensory characterization. These efforts will not only enhance consumer understanding and satisfaction, but also support innovation in breeding, cultivation, and processing.

Future research should continue to explore the interactions between compounds, the environmental factors influencing their production, and the development of preservation techniques to maintain their stability. The application of cutting-edge technologies, such as synthetic biology and computational modeling, holds promise for optimizing aroma and flavor profiles while ensuring product quality and consistency.

A comprehensive aroma wheel for *Cannabis sativa* L. products is considered desirable within the field. However, it is posited that an extensive study encompassing various strains, trained sensory panelists, and detailed metabolomic analysis is essential to ensure accurate representation. Although data obtained from producers’ descriptions offer valuable insights and serve as a general overview, it is acknowledged that such information may reflect a combination of the intrinsic characteristics of the flowers alongside commercial marketing strategies.

This review highlights the complexity and significance of cannabis aroma and flavor, emphasizing the need for continued collaboration between researchers and industry stakeholders. By addressing these challenges, the cannabis sector can unlock new opportunities for product development and scientific discovery.

## Figures and Tables

**Table 1:** Summary of key aroma and flavor terpenes in *Cannabis sativa* L., including chemical class, characteristic aroma descriptors, and flavor impressions.

Terpene	Class	Aroma Description ^1^	Flavor Impression ^1^
Myrcene	Monoterpene	Musky, earthy, fruity	Mildly sweet, herbal
α-Pinene	Monoterpene	Piney, resinous	Sharp, woody
β-Pinene	Monoterpene	Woody, turpentine-like	Resinous, slightly bitter
Limonene	Monoterpene	Citrus (lemon, orange)	Sweet, citrusy
Linalool	Monoterpene	Floral, lavender	Lightly spicy, floral
Terpinolene	Monoterpene	Floral, herbal, citrus	Mild, slightly bitter
Ocimene	Monoterpene	Sweet, herbal, woody	Subtle, slightly fruity
α-Terpinene	Monoterpene	Herbal, citrus	Mild, spicy
γ-Terpinene	Monoterpene	Citrus, lemon-like	Clean, tangy
α-Phellandrene	Monoterpene	Peppery, citrus	Pungent, slightly bitter
Sabinene	Monoterpene	Spicy, peppery	Warm, bitter
1,8-Cineole	Monoterpene	Eucalyptus, minty	Cooling, spicy
Camphene	Monoterpene	Earthy, woody	Pungent
δ-3-Carene	Monoterpene	Sweet, cedar-like	Earthy
β-Caryophyllene	Sesquiterpene	Spicy, woody, peppery	Warm, peppery
α-Humulene	Sesquiterpene	Earthy, woody	Bitter, hoppy
Farnesene	Sesquiterpene	Green apple, woody	Mild, sweet
Nerolidol	Sesquiterpene	Woody, citrusy	Subtle, bitter
Bisabolol	Sesquiterpene	Floral, sweet	Mildly bitter, soft

^1^ Classification and descriptors are synthesized from multiple sources, including Allen et al. (2019) [47], Booth et al. (2020) [5], Flores-Sanchez and Verpoorte (2008) [48], and others cited throughout Section 2.1.

## Data Availability

No new data were created or analyzed in this study. Data sharing is not applicable to this article.

## Supplementary Materials

The following supporting information can be downloaded at: https://www.mdpi.com/article/10.3390/molecules30132784/s1, Table S1: Terpene Wheel Data; Table S2: Products Data-Aroma and Terpene Tags.
